# Molecular Lesions in BRI1 and Its Orthologs in the Plant Kingdom

**DOI:** 10.3390/ijms25158111

**Published:** 2024-07-25

**Authors:** Ahmad Zada, Minghui Lv, Jia Li

**Affiliations:** 1 Ministry of Education Key Laboratory of Cell Activities and Stress Adaptations, School of Life Sciences, Lanzhou University, Lanzhou 730000, China; 2 Guangdong Provincial Key Laboratory of Plant Adaptation and Molecular Design, School of Life Sciences, Guangzhou University, Guangzhou 510006, China

**Keywords:** brassinosteroids, receptor, mutational analysis, allele, orthologs, plant varieties

## Abstract

Brassinosteroids (BRs) are an essential group of plant hormones regulating numerous aspects of plant growth, development, and stress responses. BRI1, along with its co-receptor BAK1, are involved in brassinosteroid sensing and early events in the BR signal transduction cascade. Mutational analysis of a particular gene is a powerful strategy for investigating its biochemical role. Molecular genetic studies, predominantly in *Arabidopsis thaliana*, but progressively in numerous other plants, have identified many mutants of the *BRI1* gene and its orthologs to gain insight into its structure and function. So far, the plant kingdom has identified up to 40 *bri1* alleles in Arabidopsis and up to 30 *bri1* orthologs in different plants. These alleles exhibit phenotypes that are identical in terms of development and growth. Here, we have summarized *bri1* alleles in Arabidopsis and its orthologs present in various plants including monocots and dicots. We have discussed the possible mechanism responsible for the specific allele. Finally, we have briefly debated the importance of these alleles in the research field and the agronomically valuable traits they offer to improve plant varieties.

## 1. Introduction

Plants contain numerous types of steroids; nevertheless, brassinosteroids (BRs) are widely distributed and have a biological impact on plant development when administered exogenously [1]. More than 70 chemical representatives of this class of phytohormones have been identified or isolated from more than 100 plant species, ranging from algae to angiosperms, indicating the widespread distribution across the plant kingdom [2]. BRs play many essential roles in a plant’s life cycle including the elongation, division, and differentiation of cells at the cellular level [3,4,5]. BRs control several aspects of the whole plant, including root meristem size [6,7], blooming period [8], male fertility [9], and stomata development [10,11,12]. BRs also play a variety of roles in both biotic and abiotic stress responses, including immunological signaling caused by pathogen-associated molecular patterns, thermotolerance, and defense against insect herbivores [5,13,14,15,16].

The brassinosteroid signaling pathway is one of the well-characterized pathways in plants that mediate response to BRs [17]. This pathway is initiated by a receptor complex that consists of the Leu-rich repeat (LRR) receptor-like kinase (RLK) BRASSINOSTEROID INSENSITIVE 1 (BRI1) [18] and its coreceptors from the somatic embryogenesis receptor-like kinase (SERK) family [19,20]. BRI1 belongs to a large group of serine/threonine leucine-rich repeats receptor-like kinase (S/T LRR-RLKs) exclusive to plants and has over 200 members within Arabidopsis (*Arabidopsis thaliana*) [21]. The BRI1 protein is made up of a cytoplasmic portion that includes a juxtamembrane segment (JM), a kinase domain (KD), a C-terminal tail (CT), and a single transmembrane region, while the extracellular domain (ED) contains an N-terminal signal peptide, a leucine-zipper motif, and 25 leucine-rich repeats [18,22,23]. A sequence of amino acids with 68 residues between the 21st and 22nd LRRs is referred to as an island domain (ID), and structural and genetic analyses indicate that the island domain and the 22nd LRR have a direct role in BR binding [24,25]. The BRI1 kinase activity is inhibited in the absence of BRs by its C terminus [23] and an inhibitory protein, bri1 kinase inhibitor 1 (BKI1), through physical interaction [26].

In Arabidopsis, three potential BRI1 homologs, identified as BRI1-like 1, 2, and 3 (BRL1, BRL2, and BRL3), were found with the use of bioinformatics analysis and the activation-tagging screening for *bri1* suppressors [27,28]. Additionally, BL binding studies show that BRL1 and BRL3 can bind to BL, while BRL2 does not, and BRL1 has a considerably higher binding affinity for BL than BRI1 [27]. When these homologs were expressed in Arabidopsis *bri1* mutants, BRL1 and BRL3, but not BRL2, restored their phenotypes, indicating that these two proteins have a high affinity for BL interaction [27,29].

The binding of the brassinosteroid ligand causes hetero-dimerization of BRI1 and SERK family members like BRI1-ASSOCIATED RECEPTOR KINASE 1 (BAK1) [30,31], which results in extensive auto- as well as trans-phosphorylation of the intracellular BAK1 and BRI1 kinase domains [20]. After that, the CONSTITUTIVE DIFFERENTIAL GROWTH 1 (CDG1) and the BR SIGNALING KINASES (BSKs) are phosphorylated by the activated BRI1, which subsequently phosphorylates a phosphatase termed BRI1-SUPPRESSOR 1 (BSU1) [32,33,34]. Brassinosteroid insensitive 2 (BIN2), a GSK3/Shaggy-like kinase, is dephosphorylated and inhibited by phosphorylated BSU1, which also releases its inhibition on a class of transcription factors specific to plants, including bri1-EMS suppressor 1 (BES1), brassinazole resistant 1 (BZR1), and their cofactors [35,36,37,38,39,40]. Eventually, this results in the activation of transcription factors like BES1 [40] and BZR1 [39], which cause major alterations in the expression of thousands of genes downstream [39,41]. 

Mutants that exhibit phenotypic features distinct from isogenic wild-type plants are crucial for genetic study [42]. Up to 40 distinct *bri1* alleles have been identified in Arabidopsis since the first BR-insensitive mutant was reported in 1996. The majority of these mutations are concentrated in the extracellular region’s ID or the LRRs that surround it, and the cytoplasmic region’s KD [43,44], and analyzing these mutations has improved our understanding of BRI1’s role in modulating the growth and development of plants [43]. Previous biochemical and structural analyses have revealed or implied that some *bri1* mutations impact BRI1 binding to its ligand or the coreceptor BAK1 [45,46,47,48]; some inhibit BRI1’s kinase activity [23,49,50,51]; still others prohibit BRI1 from intracellular trafficking [52,53].

In the plant kingdom, *BRI1* orthologs are widely distributed and have been isolated and characterized from a variety of species, comprising monocots like *Brachypodium distachyon*, rice (*Oryza sativa*), barley (*Hordeum vulgare*), and dicots which include tomato (*Lycopersicon esculentum*) and pea (*Pisum sativum*) [54]. In addition to the model plant Arabidopsis, in which up to 40 *bri1* alleles have been identified alone, mutations in orthologs of *BRI1* have been isolated from various plants around the plant kingdom. In rice, barley, tomato, and pea, mutations in *BRI1* orthologues lead to pleiotropic phenotypes that are alike [55,56], and similarity in gene sequences, as well as mutant phenotypes, reinforces functional conservation amongst *BRI1* genes in diverse species [54].

## 2. *bri1* Alleles in Arabidopsis

Lesions in the genes encoding the hormone receptor or components of the signal transduction system are typically the cause of hormone-insensitive mutants [57,58]. It is theoretically hypothesized that mutants that are hormone-insensitive exhibit identical phenotypes as mutants that are hormone-deficient. Actually, phenotypes of BR-deficient mutants such as *de-etiolated* (*det2*) and *constitutive photomorphogenesis and dwarfism* (*cpd*) are similar to those of dwarfed *bri1* mutants [59,60]; nevertheless, the primary distinction is that BRs do not reverse the dwarfism along with other growth defects of *bri1* mutants. In Arabidopsis, the inhibitory effect of BR on primary root elongation was critical in identifying the *bri1* mutant [61]. The recessive mutation that causes the *bri1-1* phenotype occurs in a gene situated on chromosome IV [61]. The gene impaired in BR-insensitive mutants was identified and found to encode a potential membrane-bound LRR-RLK [18]. The majority of the alleles with the most severe morphological abnormalities have point mutations within the island or kinase domain [49]. To some extent, these phenotypes exhibit similar characteristics, such as dwarfism, compact, dark-green rosette leaves with short petioles, delayed senescence and flowering time, decreased male fertility, and modified skotomorphogenesis and photomorphogenesis [61,62]. To date, up to 40 unique *bri1* alleles have been identified in Arabidopsis during the last three decades by several independent genetic screens (Table 1).

### 2.1. A BR-Insensitive Mutant Shows Multiple Defects 

The first BR-insensitive mutant identified in Arabidopsis was *bri1* [61]. Mutation within the *BRASSINOSTEROID-INSENSITIVE 1* (*BRI1*) locus significantly impacts plant development, suggesting that the *BRI1* gene has a potential function in brassinosteroid sensing or signal transduction. The mutant was originally named *brassinosteroid-insensitive 1 (bri1)*; later, it was renamed *bri1-1*. *bri1-1* did not respond to BRs in root-inhibition assays, but retained sensitivity to other plant hormones such as auxin and gibberellin (GA). While Col-0 plants of the same age were taller than 15 cm, two-month-old *bri1-1* plants were merely 1.5 cm tall. It is quite likely that the *bri1-1* mutant is female-fertile while male-sterile. There is only one recessive Mendelian allele responsible for the *bril-1* phenotype. The *bri1-1* phenotype, on the other hand, was revealed to be firmly linked to the bottom of chromosome IV. The *bri1-1* mutant exhibited several defects in developmental pathways that were not remedied by brassinosteroid treatment. These included a significantly reduced stature; thickened, dark-green leaves; male sterility; and decreased apical dominance. When the *bri1-1* mutant was grown in the dark air, it exhibited short, thickened hypocotyls and entirely opened cotyledons, which are traits shared by the *det2* and *cop* class of photomorphogenic mutants [76,77]. Microscopic assessment of the *bri1-1* mutant suggests that reduced cell size rather than cell number is liable for the dwarfed phenotype.

### 2.2. Brassinosteroids Are Essential in Plant Development 

A brassinosteroid-insensitive mutant called *cbb2* (*cabbage 2*) was analyzed, which was allelic to *bri1-1* [63]. Through the characterization of the mutant, the study provided convincing evidence that this class of compounds (brassinosteroids) are vital as endogenous regulators of cell elongation and play a significant role in plant growth. Extremely dwarfed mutant *cbb2* was isolated by transposon mutagenesis in *Arabidopsis thaliana* [78]; *cbb2* mutation disturbs the perception of brassinosteroids, which are essential for properly regulating plant development. Dark-grown *cbb2* seedlings exhibited characteristics similar to those seen in *de-etiolated* (*det*) [76] or *constitutive photomorphogenesis* (*cop*) plants [77] which included short hypocotyl, opened cotyledons, and the appearance of primary leaves after sustained growth in darkness. These differences were observed in comparison to wild-type plants, which developed extended hypocotyl and small, closed cotyledons in dark conditions [79]. Similar to *bri1-1*, the mutant seedlings’ smaller size was typically brought about by smaller cells, as opposed to fewer cells overall. Molecular genetic markers aided with the mapping of the *cbb2* mutation to the basal end of the fourth chromosome.

### 2.3. An LRR Receptor-like Kinase Is Involved in BR Signaling Pathway

**Figure 1:**
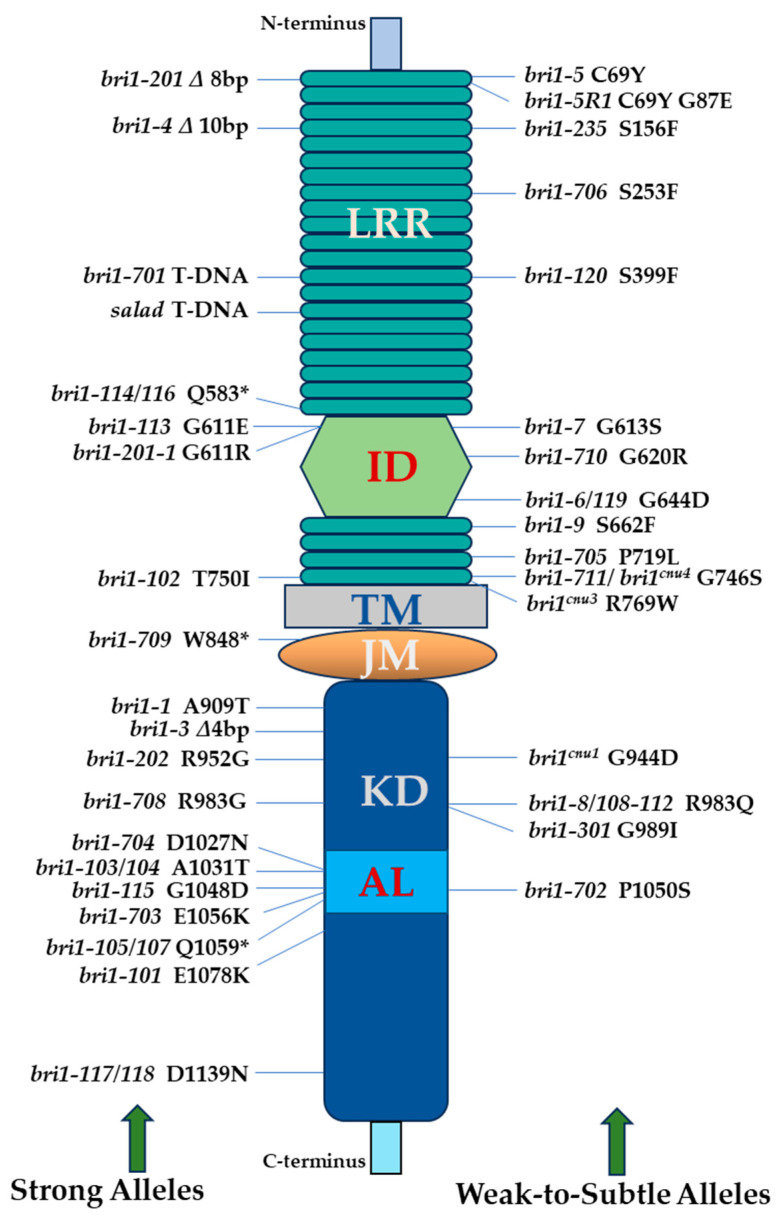
Diagram showing *bri1* alleles in Arabidopsis identified so far; for references, see Table 1. Leucine-rich repeats (LRRs), island domain (ID), transmembrane (TM), juxtamembrane (JM), kinase domain (KD), and activation loop (AL). ∆ represents deletion, * represents stop codon.

The cloning and molecular analysis of the *BRI1* gene’s expression pattern in 1997 marked an important breakthrough in the field of brassinosteroid research. The *BRI1* gene encodes a putative receptor-like kinase expressed everywhere in plants and is involved in the BR signaling pathway [18]. *BRI1* appears as being constitutively and ubiquitously expressed in response to varying light conditions and throughout the development of Arabidopsis. In this study, 18 *bri1* alleles were isolated of which 5 were sequenced and characterized. These eighteen mutations were alleles of the formerly identified *BRI1/CBB2* gene [61,63], and were labeled *bri1-101* to *bri1-118*, as these were the alleles of the *bri1* locus that had previously been reported. The *bri1* mutants exhibited several characteristics in the dark, including dwarfed stature, thickened hypocotyls, accumulated anthocyanin, opened and enlarged cotyledons, and developed primary leaf buds. The mutants displayed reduced male fertility and apical dominance, delay in flowering, and leaf senescence. Out of 18 alleles, 5 alleles that were sequenced, a mutation in the extracellular domain, and 4 other mutations in the cytoplasmic kinase domain abolish the in vivo activity of BRI1 (Figure 1). Four alleles with mutations in the kinase domain are grouped in a 50-amino acid stretch (amino acids 1031–1080). A premature termination caused by a nonsense mutation at codon 1059 in *bri1-107* results in the deletion of 138 amino acids at the C terminus, suggesting a polypeptide lacking the final three subdomains of the kinase domain. Conserved residues have been substituted in two more alleles. In subdomain VIII, *bri1-115* carries a mutation that results in aspartate instead of conserved glycine. A mutation in codon 1078 of *bri1-101* causes a positively charged lysine to replace a conserved negatively charged glutamate in subdomain IX; glutamate at this position is completely conserved in plants through the LRR receptor kinase family. At codon 1031, the second residue just after the conserved DFG triplet in subdomain VII, which is involved in ATP binding, *bri1-104* converts an alanine to threonine. Nonetheless, the island domain is necessary for BRI1’s function in the transduction of brassinosteroid signals. Gly-611 has been altered to a negatively charged glutamate in the severe allele *bri1-113* in the island domain.

### 2.4. BRI1 Is Involved in BR Homeostasis

Seven more *bri1* alleles, ranging from nulls to weak alleles were identified and characterized in a study that further demonstrated that brassinosteroids-insensitive dwarf mutants accumulate large quantities of BRs, and for endogenous BR levels to remain in a homeostatic state, *BRI1* is necessary [64]. Based on their physical traits, these seven *bri1* mutants can be classified as severe, moderate, or weak alleles (Figure 1). The severe alleles *bri1-3* and *bri1-4* are characterized by their small size, all main above-ground organs, dark-green color, and rare seed production. There is an association between decreased height and infertility of *bri1* mutants. It is anticipated that the small deletions in *bri1-3* and *bri1-4* will change the BRI1 open reading frame (ORF) and produce an early stop codon that represents null alleles of *bri1*. A single base-pair change that resulted in an amino acid alteration was present in both the weak and intermediate alleles. These mutations were dispersed across the *BRI1* gene: four of them were found in the extracellular domains, and one (*bri1-8*) changed a conserved residue in the cytoplasmic kinase domain. The *bri1-8* intermediate variant produces more fertile and slightly bigger plants than the *bri1-3* and *bri1-4* alleles. The phenotype of *bri1-8*, however, is not as strong as would have been anticipated for a mutation in such a conserved residue, seen in numerous RLKs [18,80]. The weak group of *bri1* alleles consists of four alleles; despite being shorter in stature, plants of the four weak alleles have colors similar to the wild type and are somewhat fertile, albeit not as fertile as the wild type. Gly alterations occurred in a region of the island domain between two LRRs in two different mutations (*bri1-6* and *bri1-7*). In the island domain, Li and Chory [18] reported a mutant containing a different Gly; nevertheless, their mutant was not fertile, suggesting that it was likely a severe allele. The two remaining mutations were found in the extracellular domain: a Ser to a Phe in the first LRR following the island domain (*bri1-9*) and a Cys to a Tyr in the paired Cys domains found in the amino terminus of the extracellular domain (*bri1-5*). The first known mutations in the extracellular region of BRI1 that are not in the island domain were these two weak alleles. It is interesting to note that every missense mutation identified within the extracellular domain in this study is a weak allele, and that, with a few notable exceptions, these mutants exhibit proportionate changes in the plant’s physical characteristics. The *bri1-5* mutant has wider leaves than the wild type, and their internode distance is shorter than that of other *bri1* weak alleles, such as *bri1-7*, even though *bri1-5* plants are taller than *bri1-7* plants. Brassinolide, a physiologically active BR for Arabidopsis growth, accumulates in *bri1* mutants. The proportion of brassinolide for the wild type (Ws-2), a weaker allele (*bri1-5*), and a null allele (*bri1-4*), was 1:22:57. Therefore, the quantity of functional BRI1 protein is correlated with the accumulation of brassinolide. The *BRI1* gene is necessary for the feedback regulation of BR biosynthesis, because both a null allele and a weak allele of *bri1* exhibit a significant accumulation of brassinolide, along with other intermediates.

### 2.5. BRI1 Is Ubiquitously Expressed

Another research study revealed the isolation and molecular characterization of five additional novel *bri1* alleles and demonstrated that BRI1 is a ubiquitously expressed leucine-rich receptor involved in the BR signaling pathway via phosphorylation of Ser/Thr [49]. Out of the five alleles (Figure 1), three mutations were found in the kinase domain; *bri1-1* and *bri1-108* are missense alleles that change the amino acids Ala-909 to Thr and Arg-983 to Gln, respectively. All protein kinases have Ala-909 in subdomain II, while Arg-983 in subdomain VIA is conserved among potential plant LRR receptor-like kinases such as BRI1, CLV1, ERECTA, and Xa21 [18]. A non-conserved Asp to Asn mutation at codon 1,139, in a region of the protein that typically comprises negatively charged residues in protein kinases, is present in the *bri1-117* allele. The importance of the BRI1 kinase domain is once again shown by these mutations. Two mutations were found in the BRI1 extracellular domain: a missense mutation in *bri1-102* leads to the replacement of an Ile for Thr-750, and a nonsense mutation in *bri1-114* creates a stop codon earlier within the 70-amino acid island domain. The kinase activity of both the wild-type and bri1-113 proteins is capable of autophosphorylation, while the kinase activity of bri1-101, which has a mutation in the kinase domain, is substantially decreased.

### 2.6. Interaction between BR and GA in Plant Development 

A research investigation identified and analyzed the *bri1* allele *bri1-201* from gamma-mutagenized plants and subsequently highlighted the fact that two important plant hormones, BR and GA, interact with each other in plant development; nevertheless, certain such interactions are antagonistic [67]. *bri1-201* is an entirely novel deletion allele of the *BRI1* gene expressing the BR receptor BRI1, as determined by map-based cloning and genome sequencing [18,45,49]. The mutant displayed reduced growth at the initial stages of development, decreased apical dominance, late flowering, senescence of leaves, and male sterility. According to physiological studies, *bri1-201* is not responsive to BRs. *bri1-201* was discovered to have an 8-bp deletion of 22 nucleotides downstream of the start codon ATG (Figure 1), which caused a frameshift in the ORF, ending in a stop codon after 44 amino acid residues. Due to its absence of the BRI1 protein and somewhat smaller stature than *bri1*, *bri1-201* is considered a null allele that is identical to the deletion mutant *bri1-4*, which displays a frameshift at 140 amino acid residues in the BRI1 open reading frame [64].

### 2.7. BRs Are Involved in Flowering Time in Arabidopsis 

Two alleles of *bri1*, as strong enhancers of the autonomous mutant *luminidependens* (*ld*), were isolated and molecularly characterized in a study, which subsequently provided evidence that BRs are involved in floral timings by regulating *FLOWERING LOCUS C* (*FLC*) expression [8]. Both of these alleles were mapped to the *BRI1* locus and were named *bri1-201* and *bri1-202.* A mutant allele with the name *bri1-201* has already been reported by Bouquin et al. [67]; hence, for convenience in this review, we renamed the mutant *bri1-201* as *bri1-201-1*. It was revealed that the isolated alleles, *bri1-201-1* and *bri1-202*, had point mutations that impacted the kinase and BR-binding domains, respectively (Figure 1). BR-deficient mutants and *bri1* mutants displayed slightly late flowering, whereas the *bas1 sob7* double mutant showed minor early flowering due to its decreased ability to metabolize BRs to their inactive forms [18,59,81,82]. The severe phenotype of the double mutants *cpd ld* and *bri1 ld* led to the observation that BR activity is necessary for floral transition to occur at the proper time.

### 2.8. Is the Kinase Activity of BRI1 Essential for Its Function?

Isolation and molecular characterization of a novel allele of *bri1*, *bri1-301,* shows moderate morphological phenotypes and a reduced response to BRs under normal growth conditions [70]. The conversion of Gly to Ile of codon 989 in the VIa subdomain of the kinase domain of BRI1 is due to a two-base alteration from GG to AT (Figure 1). The amino acid substitution resulted in undetectable kinase activity of bri1-301 protein, and raises questions regarding the functional necessity of BRI1 kinase activity in plant growth and development. With almost normal fertility, the mutant had mild morphological defects such as round leaves, short petioles, a longer life span, and somewhat lower plant height. The only two weak *bri1* alleles known to exist that have mutations in the BRI1 kinase domain were *bri1-8* and *bri1-301* [64], until *bri-702* was identified in 2017 [51]. Two independent studies revealed that *bri1-301* is a temperature-sensitive mutant [69,71]. High temperature reduced the protein stability and biochemical activity of bri1-301, likely due to temperature-enhanced protein misfolding leading to its increased internalization and degradation [69,71]. At a lower temperature such as 18 °C, *bri1-301* displays subtle morphological abnormalities, while at a higher temperature such as 28 °C, *bri1-301* shows a severe phenotype resembling that of a null *bri1* mutant [69]. In this study, the importance of the BRI1 kinase activity was questioned [70] because bri1-301-KD showed no kinase activity in vitro; however, Sun et al. [51] showed that bri1-301-CD possesses kinase activity for its substrate BAK1 in vivo. Zhang et al. [71] further confirmed that the bri1-301 mutant receptor exhibits weak BR-triggered phosphorylation in vivo and absolutely entails its kinase activity for the partial growth that happens in the *bri1-301* mutant.

### 2.9. T-DNA Insertion bri1 Alleles

A unique T-DNA insertion mutant of *BRI1* named *salade* was identified in a study that further demonstrated that T-DNA insertion mutagenesis is a powerful technique for the functional analysis of genes in plants [72]. The phenotypic similarities between the *salade* plants and robust loss-of-function *bri1* mutants suggest that the BRI1 deletion is the primary cause of their morphologies. Several *bri1* alleles had already been studied in detail, and the strong loss-of-function mutants had dwarf phenotypes that were very similar to those of the mutant *salade* [18,61,64]. Two T-DNAs are present in the *salade* genome; one is inserted 2885 bp upstream of the *BRI1* start codon, and the other 749 bp upstream (Figure 1). Wild-type plants of the same age were taller than 30 cm, but two-month-old *salade* plants could not become higher than 2 cm. In addition to male sterility and leaf senescence, the salade plants showed delayed flowering.

Gou et al. [73] characterized a novel T-DNA insertional allele of *bri1* named *bri1-701* from the seeds ordered from ABRC. *bri1-701* showed no full-length mRNA expression and exhibited a phenotype identical to a typical null *bri1* mutant. The root and hypocotyl growth of *bri1-701* was completely insensitive to BL, and it is a T-DNA insertion strong knockout allele in the extracellular LRRs of *BRI1*. After the isolation of *bri1-701*, it has been extensively used in molecular genetic studies.

### 2.10. Intragenic Suppressor of bri1-5

Characterization of a dominant suppressor of the dwarf phenotype of *bri1-5* plants revealed that suppression is caused by a second-site mutation in *BRI1*, *bri1-5R1* [65]. The study put forward a model in which the second point mutation *bri1-R1* compensates for the abnormalities caused by protein folding in *bri1-5* and restores BRI1’s accumulation, along with plasma membrane localization, to a certain level. In terms of certain features such as plant height, spacing among internodes, silique length, and number of seeds per seed pod, *bri1-5R1* plants were generally intermediate between *bri1-5* and Ws-2 plants. Along with the *bri1-5* mutation, the first LRR (G260A, Gly87Glu) has been mutated in the sequenced *bri1-5R1* plants (Figure 1). The glycine that was changed in bri1-5R1 is found in the LRRs of BRI1, as well as in other model RLKs, in a highly conserved manner. The weak mutant *bri1-9*, brought on by a missense mutation located in the 22nd LRR, is the only other LRR missense mutation found in *BRI1* [64]. Therefore, the second mutation discovered in the LRRs of BRI1 via forward genetics is *bri1-R1*. *bri1-5R1* plants have distinct phenotypes as a result of both enhanced cell expansion and higher cell division. 

### 2.11. LRR Domain of BRI1 Is Necessary

A natural *bri1* allele, named *bri1-120* in Landsberg (Ler) background was molecularly characterized, the mutant displayed defective growth and reduced BR sensitivity [66]. This was a new allele of *bri1* in the LLR region before the island domain, and the study showed that the LLR domain is necessary for the biological function of BRI1. The mutant’s phenotypic characteristics, such as its restricted growth, dark-green compact rosette leaves, and downward curling, gave an appearance that it was a weak *bri1* mutant like *bri1-301* [70]. Due to a nucleotide mutation (T to C) at the 1196th position, *bri1-120* has phenylalanine at the 399th position in the 13th LRR, rather than serine (Figure 1). This particular LRR area is essential for normal BRI1 functioning, based on studies investigating a point mutation in the 13th LRR, which is located before the 70-amino-acid island section of the extracellular domain of BRI1.

The isolation and characterization of a loss-of-function mutant *bri1-235* that carries a mutation in the less-conserved fourth LRR of the BRI1 extracellular domain in Arabidopsis provides insight into the importance of BRI1’s less-conserved LRRs [68]. The research report also demonstrates that for BRI1 folding, not just the island and 13–25th LRRs, but the initial few LRRs following the signal peptide are, likewise, crucial. According to DNA sequencing analysis, the *bri1* allele, *bri1-235*, had a single base change from C to T in the fourth LRRs of BRI1; as a result, the 156th amino acid residue of BRI1 in the *bri1-235* changed from serine to phenylalanine (Figure 1). The mutant plants were smaller than usual and had spherical leaves, short petioles, a longer life span, smaller rosettes, and roughly normal fertility in the presence of light. Two ER-retaining *bri1* mutants, *bri1-5* and *bri1-9,* have mutations in the LRR of the BRI1 extracellular domain [52,53,64]; *bri1-235* is a mutant that is identical to the *bri1-5* or *bri1-9* mutants in terms of the mislocalization of the receptor. 

### 2.12. Suppressors of PMEIox

Wolf et al. [75] reported the isolation of the *comfortably numb1* (*cnu1*) mutant, in which most aspects of the *PMEIox* (overexpressing a pectin methylesterase—a PME inhibitor protein referred to as PMEIox) phenotype were suppressed. Genetic mapping revealed that the kinase domain of the brassinosteroid (BR) receptor BRI1 contains a mutation (G944D), which leads to the suppressor phenotype (Figure 1). The mutant allele was named *bri1^cnu1^*, and adult *bri1^cnu1^* plants had a typical BR-deficient phenotype of moderate strength compared to published *bri1* mutants. 

In addition to *bri1^cnu1^*, two extragenic suppressor mutants called *bri1^cnu3^* and *bri1^cnu4^* strongly suppressed the macroscopic *PMEIox* growth phenotype in seedlings [74]. The research highlights the complex nature of plant plasma-membrane receptor function and offers an approach to distinguish between BRI1’s roles in BR signaling and its noncanonical functions. It was revealed that a mutation in *bri1^cnu3^* caused Arg 769, which is located in the region closest to the extracellular membrane, to be exchanged for Trp (R769W), while a mutation in *bri1^cnu4^* caused Gly 746, which is located in the final LRR repeat of the extracellular domain, to be replaced by Ser (G746S) (Figure 1). Both mutants displayed rather normal development and did not significantly differ from the wild type, in contrast to the previously found *PMEIox*-suppressing mutant *bri1^cnu1^* [75]. Therefore, compared to *bri1^cnu1^*, which carries a mutation in the kinase domain, *bri1^cnu3,^* and *bri1^cnu4^* are two weak *BRI1* mutants with a modest growth phenotype, most probably because of mutations in a less-conserved extracellular region of *BRI1*. It is interesting to note that in a TILLING technique for acquiring additional *bri1* mutants, a similar mutation described here as *bri1^cnu4^*, G746A (G2236A on a nucleic acid level), has been identified as *bri1-711* [51]. Both *bri1^cnu4^* and *bri1-711* displayed moderate insensitivity to exogenous BL treatment, as well as minor growth abnormalities.

### 2.13. Additional bri1 Alleles via TILLING Analysis

Using the Targeted Induced Local Lesions in Genomes (TILLING) strategy, nine alleles of *bri1* were isolated and characterized including four subtle, one weak, and four strong [51]. These alleles provide additional insight into the comprehension of early events in the BR signaling pathway. These mutants were named in the order of their identification, from *bri1-702* to *bri1-711* (Figure 1). Like *bri1-5*, *bri1-9*, and *bri1-301*, *bri1-702* displays a weak phenotype of *bri1* [64,70]. *bri1-705*, *bri1-706*, *bri1-710*, and *bri1-711*, in comparison, have even milder phenotypes than *bri1-702* and were thus designated as subtle alleles. However, *bri1-703*, *bri1-704*, *bri1-708*, and *bri1-709* exhibit severe phenotypes that are similar to *bri1-701*, a previously reported *bri1* null allele [73]. It is interesting to note that *bri1-707* and *bri1-301* share a mutation in Gly-989, although they differ in the amino acid exchange. Consequently, Xu et al. [70] reported that *bri1-301* has a weak *bri1* phenotype, but *bri1-707* does not display an obvious defective phenotype. Similarly, discrete substitutions at the same residue are present in *bri1-708* and *bri1-8/108-112*; while *bri1-708* exhibits a null *bri1* phenotype, *bri1-8*/*108-112* are intermediate *bri1* mutants [64]. *bri1-702*, which is the only weak allele in BRI1’s activation loop, carries an alteration in Pro-1050, which is well conserved in BRI1. 

## 3. Orthologs of *bri1* Allele

### 3.1. lka Mutant of Garden Pea

**Figure 2:**
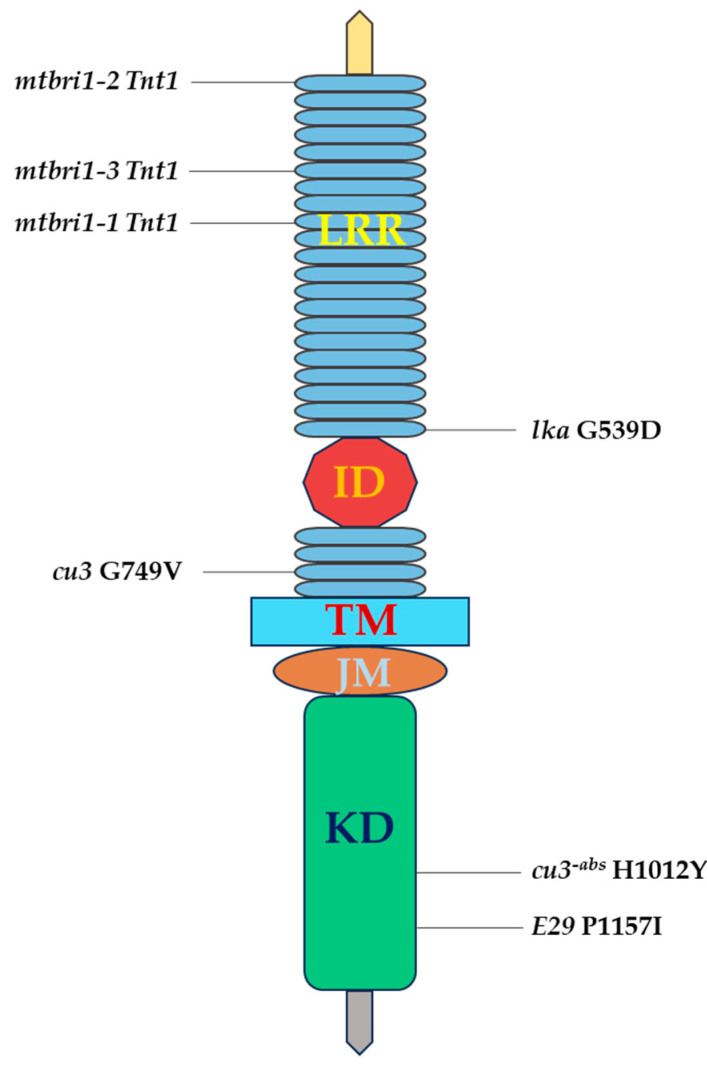
Diagram showing mutation sites in *BRI1* orthologs of tomato, pea, pepper, and M. *truncatula.* Leucine-rich repeats (LRRs), island domain (ID), transmembrane (TM), juxtamembrane (JM), and kinase domain (KD).

Nomura et al. [83] observed that the garden pea (*Pisum sativum*) dwarf mutant *lka* is around a hundredfold less sensitive to brassinolide as compared to the *lkb* mutant, indicating that the *lka* lesion causes decreased sensitivity to BR. Application of exogenous BRs restored the *lkb* mutant, which was BR-deficient, to a normal height; it had only a minimal effect on *lka* plants. In dark conditions, *lka* plants do not display the de-etiolation traits observed in Arabidopsis mutants such as *det2* and *cpd*. [59,60]. Later, Nomura et al. [83,84] reported that the dwarf mutant *lka* of garden pea is less sensitive to brassinolide, due to a point mutation in the dwarf *lka* pea plant’s *LKA* gene, a garden pea homolog of *BRI1* also known as *PsBRI1*. The dwarfism of the *lka* mutant was characterized and shown to be caused by a nucleotide substitution (G1690A) in the LRR next to the N-terminus of the island domain and is projected to transform aspartic acid to D564N asparagine (Figure 2) [85]. The *lka* mutation appears to have a significant impact on brassinolide perception, based on its close proximity to the amino acid island in *PsBRI1* and its corresponding reduction in BR sensitivity. The *lka* mutant displayed a limited sensitivity to brassinolide, however, because *lka* is not a null mutation.

### 3.2. cu3 Alleles of Tomato

A naturally occurring tomato dwarf mutant *cu3* was discovered among seedlings of *L. pimpinellifolium*. Koka et al. [86] showed that this recessive, single-gene mutant exhibits numerous traits similar to the Arabidopsis BR-insensitive mutant *bri1* [61], such as extreme dwarfism, curled leaves with a dark-green color, delayed development, and decreased fertility. The *cu3* mutant plants exhibited sensitivity to gibberellin, abscisic acid, cytokinins, and other hormones, but were insensitive to brassinolide. Similar to *bri1*, *cu3* is an extreme dwarf that has a maximum mass of 2.5 cm in all dimensions. Later on, the *cu3* mutant was further characterized by Montoya and colleagues [87]. 

A dwarf mutant of tomato was observed to be partially sensitive to BL [87], and hence the mutant appeared to be different from the previously reported BR-insensitive mutant *curl3* (*cu3*) [86]; therefore, initially, it was named *altered brassinolide sensitivity1* (*abs1*). Using degenerate primers, the tomato counterpart of the Arabidopsis *Brassinosteroid Insensitive1* Leu-rich repeat (LRR) receptor-like kinase, known as *tBRI1*, was identified [87]. Allelism tests revealed that *cu3* and *abs1* were allelic; *abs1* represents a weak (intermediate) recessive allele situated at the *cu3* locus, and, thus, the *abs1* allele of *cu3* was termed as *cu3^-abs^* or the *abs1*. Sequence comparison demonstrated the presence of G749Z, a nonsense mutation, in *cu3* (Figure 2). A missense mutation within the kinase domain, H1012Y, has been found in the *cu3^-abs^* mutant, indicating that *cu3* is a null allele and that *cu3^-abs^* might possess tBRI1 activity and potentially produce a weak allele (Figure 2). The mutant plants that carry the weak *cu3^-abs^* gene are 50% as tall as plants of the wild type, whereas *cu3* mutants, on the other hand, are just 5% of the height of the wild type, and the stop codon indicates that *cu3* is a null allele.

### 3.3. d61 Alleles of Rice

**Figure 3:**
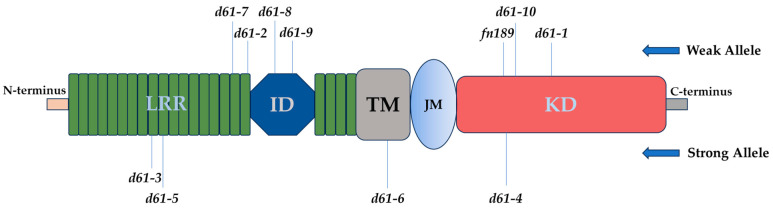
Diagram showing *d61* alleles in rice. Leucine-rich repeats (LRRs) island domain (ID), transmembrane (TM), juxtamembrane (JM), and kinase domain (KD).

The dwarf mutant *d61*, which is defective within the rice *BRASSINOSTEROID INSENSITIVE 1* (*BRI1*) gene expressing for OsBRI1, the BR receptor kinase, was, in fact, the first known BR-related mutant in rice (*Oryza sativa*) [55]. Later, a series of *d61* alleles of rice were identified, carrying mutations in several domains of the *OsBRI1* gene (Table 2). Loss-of-function mutations result in semi-dwarfism, erect height, BR insensitivity, and occasionally sterility. The *d61* mutants exhibit varying degrees of growth defects, and an erect phenotype [55,88,89]. To date, more than ten unique *d61* alleles of the *OsBRI1* gene have been identified, and these mutations cause changes in various domains of the OsBRI1 protein (Figure 3). Yamamuro et al. [55] isolated and characterized the *OsBRI1* gene and showed that it is quite similar to the Arabidopsis *BRI1* gene throughout its length. Many domains found in Arabidopsis BRI1 are likewise present in the anticipated OsBRI1 polypeptide; Li and Chory [18] discussed the functions of these domains. These consist of a putative signal peptide, two cysteine pairs that are spaced conservatively, a transmembrane domain (TM), a leucine-rich repeats (LRRs) domain, and a kinase domain (KD). OsBRI1 lacks three LRR domains, which corresponds to the third-to-fifth Arabidopsis BRI1 repeats when compared to the BRI1 sequence. 

The phenotypical and molecular characterization of two rice dwarf mutants, *d61-1* and *d61-2*, revealed that dwarf mutants were caused by alleles from a single locus that remained less sensitive to BR compared to the wild-type [55]. Initially, these were classified as two distinct mutants, due to the significantly shorter culm of *d61-2* compared to *d61-1*. Furthermore, *d61-1* displays the internode elongation pattern of the dm-type, while *d61-2* displays the d6-type [91]. The lack of function of the Arabidopsis *BRI1* gene’s rice counterpart results in the *d61* mutation. A single nucleotide mutation (C to T in *d61-1* and G to A in *d61-2*) at distinct positions may be found in each mutant allele when comparing the *OsBRI1* sequences of the wild type and the *d61-1* and *d61-2* mutants. In the kinase domain of OsBRI1 at residue 989, threonine changes to isoleucine as a result of the *d61-1* mutation. There is a change of valine to methionine at residue 491 of the 17th LRR in the *d61-2* just before the 70-amino-acid island domain.

In addition to the previously identified spontaneous mutants *d61-1* and *d61-2*, Nakamura et al. [89] isolated eight additional alleles, including null mutations, of the *OsBRI1*. These ten mutations were identified in four different domains: the transmembrane domain (one allele), the kinase domain (three alleles), the 70-amino-acid island (ID; two alleles), and the leucine-rich repeats (LRRs; four alleles). The single nucleotide substitutions in the mutants *d61-4* and *d61-6* resulted in a stop codon at Glu-847 and a 2-bp insertion at Asp-759, which caused a frameshift, respectively. It was therefore predicted that these mutants might have displayed the most severe phenotypes and that they would have lost OsBRI1 function. The alleles responsible for single nucleotide variations at LRR, *d61-3*, *d61-5*, *d61-7*, and *d61-2* exhibited variable degrees of phenotypic severity. Two severe alleles, *d61-3* and *d61-5*, were anticipated to have amino acid alterations that would disrupt either the secondary structure, the tertiary structure, or both, of the LRR, and severely affect its function. A Val residue that was replaced with a Met one in the LRR directly in front of the ID was linked to the intermediate phenotype *d61-2*. In ID, the mutations that caused *d61-8* and *d61-9* were situated at Gly-522 and Gly-539, respectively; even though ID has been linked to BR binding, none of the mutants showed a severe phenotype. The Arabidopsis *bri1* mutation *bri1-113*, whose mutation location coincides with that of *d61-9*, gives a severe phenotype [18]. The phenotypic intensity of the *bri1* mutant in Arabidopsis is correlated with its ecotype [27]. Hence, the severity *bri1-113* mutant of the Arabidopsis might be influenced by its ecotype. While BRI1 function should require kinase activity, nevertheless, amino acid substitutions of mutant *d61-10* and *d61-1* within the kinase domain did not result in major defects in BRI1 function. This is likely because the kinase activity of BRI1 was not affected by the amino acid exchanges and it is believed that these amino acid residues have no significance for kinase activity [22,23]. Despite having normal pattern development and differentiation, the most severe mutant, *d61-4*, displayed extreme dwarfism and twisted leaves. Cell-elongation defects and cell-division disruptions following cell-fate determination were the primary causes of this severe shoot phenotype. The *d61-4* mutant exhibited a modest root phenotype in contrast to its severe shoot phenotype. Morinaka et al. [88] selected *d61-7* out of these *d61* alleles of *OsBRI1*, due to agronomically useful traits such as semi-dwarf stature, erect leaves, and elongated neck internodes for further analysis of grain production in paddy fields, which will be discussed in the later section.

Molecular characterization of classic rice mutant *Fn189* with a semi-dwarf stature and erect leaves [90], revealed that the mutant had the same defective phenotype as the BR-deficient *d2* or BR-insensitive *d61* mutants [55,92]. The study showed that for brassinosteroids to maintain normal plant growth and development in rice, OsBRI1 kinase activity is necessary. Compared to wild-type seedlings, *Fn189* mutant seedlings showed reduced sensitivity to exogenous BL. Sequencing analysis showed that *Fn189* is a novel allelic mutant of *D61*, and found that the 2500th base within the coding area of *D61* was altered from A to T. In the kinase domain of OsBRI1, this mutation resulted in the 834th amino acid being changed from isoleucine (I) to phenylalanine (F). Since the mutant residue I834 in *Fn189* is highly conserved across BRI1-related proteins, the OsBRI1 mutant’s kinase domain I834F substitution significantly reduced OsBRI1’s kinase activity. Phenylalanine is an aromatic amino acid with fairly different physical and chemical properties, replacing isoleucine, which may lead to conformational changes in the kinase domain, resulting in its reduced activity.

### 3.4. uzu Alleles of Barley

**Figure 4:**
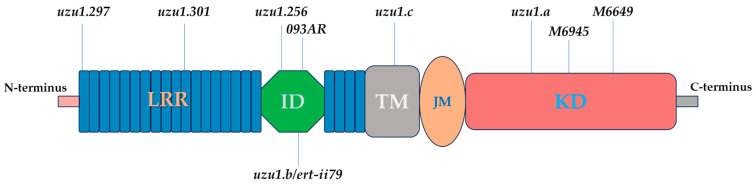
Diagram showing *uzu1* alleles in barley. Leucine-rich repeats (LLRs), island domain (ID), transmembrane (TM), juxtamembrane (JM), and kinase domain (KD).

With the use of forward and reverse genetics, several *uzu1* alleles of the *BRI1* homologous gene, *HvBRI1*, have been determined in barley (*Hordeum vulgare*) [93,94,95]. After cloning the *HvBRI1* gene, Chono et al. [93] demonstrated that the putative HvBRI1 polypeptide had a conserved signal peptide, an LRR domain with a 70-amino-acid island, a transmembrane domain, and a kinase domain, which are conserved among BRI1 homologs. There are 22 tandem copies of LRR in the LRR domain, and the copy number of LRR in HvBRI1 is similar to that of the homolog of rice BRI1. In contrast to the homologs of Arabidopsis, tomato, and pea, the rice and barley BRI1 homologs lack three copies of LRR. The mutations are localized in various fragments of the intronless gene and induce the substitution of amino acid residues positioned in distinct functional domains of the encoded BR receptor (Figure 4) [94].

A semi-dwarf barley that has the “*uzu*” gene, known as “uzu” barley in Japan, was found not to show any response to exogenously applied BL [93]. To verify if *uzu* barley possesses the mutation(s) within the barley *BRI1* gene (named *HvBRI1*), the homolog *BRI1* segment with 3558-bp was isolated from both normal and *uzu* barley, and was sequenced. A single nucleotide alteration (A-2612 to G-2612) is associated with the *uzu* phenotype, according to a comparison of the *HvBRI1* sequences in *uzu* and normal-barley varieties. In subdomain IV of the kinase domain, the alteration in *uzu* barley causes the conversion of His (CAC) to Arg (CGC) at position 857. The *uzu* dark-grown seedling did not exhibit a true de-etiolated phenotype in the dark and is not a typical de-etiolated mutant like BR-deficient *dumpy* [86]. There is uniformity in the accumulation pattern of BR in *uzu* and other BR-insensitive mutants [55,64,83,87]. The agronomical importance of the dwarf *uzu* allele will be discussed in the later section. 

The phenotype of brassinosteroid-insensitive, semi-dwarf barley mutant *093AR*, which originated from European spring barley, was allelic to the natural mutant *uzu*, and it contains an alteration in the *HvBRI1* gene [95]. The phenotype of the mutant *093AR* is caused by double substitutions CC>AA found in the *HvBRI1* gene, which induces the mutation of highly conserved threonine-573. The substitution of a basic lysine for the hydrophilic threonine-573 could cause the polypeptide to alter in conformation and, consequently, affect its catalytic effectiveness. Despite having amino acid substitutions in different HvBRI1 receptor domains, the barley mutants *uzu* and *093AR* are fully fertile and exhibit very similar phenotypes, including reduced plant height, lack of enhanced seedling elongation during dark-adapted growth, and lack of response to varying concentrations of exogenously employed BL.

Additional alleles of the barley *BRI1* gene *HvBRI1* were isolated and characterized [94]. Sequence analysis of *HvBRI1* showed mutations in *uzu1.256* (A1733T) and *uzu1.b*/*ert ii.79* (C1760A and C1761A). The region coding the steroid-interaction island domain contains the amino acid residues that are obstructed by the alterations in *uzu1.256* (Arg-564 to Trp) and *uzu1.b* (Thr-573 to Lys). The steroid binding and the overall location of the brassinosteroid binding site may be impacted by the charged Lys-573 that surrounds the hydrophobic active site and destroys charge neutrality. The C lobe of the N-terminal cap contains the amino acid substitution (Phe-53 to Ser) that was found in *uzu1.297* [25]. BRI1 may be retained in the endoplasmic reticulum due to structural instability caused by Ser-53 substitution [53]. The substitution of the nonpolar Val-282 residue for the negatively charged Asp in *uzu1.301* leads to the creation of a salt bridge among Asp-282 and Lys-302, which may disrupt the folded protein structure due to Lys-302’s loss of solvation energy [96]. An additional novel *HvBRI1* allele is *uzu1.c*, a single-nucleotide G2171A mutation that generates a weak brassinosteroid phenotype by substituting Lys for the semi-conserved Arg-710 within proximity of the BRI1 transmembrane binding domain. 

The molecular cloning and fine-mapping of a semi-dwarf gene derived from the Chinese cultivar ‘TX9425’, in two-row barley revealed the same mutation as *uzu* barley [97]. Some traits common to so-called *uzu* barley are displayed by the two-rowed dwarf barley landrace ‘TX9425,’ including the distinctive elongation of coleoptiles, leaves, culms, rachis internodes, awns, glumes, and kernels. The tall and dwarf NILs (near-isogenic lines) had identical *HvBRI1* sequences according to sequence comparisons, except the semi-dwarf allele had a single nucleotide substitution (A-2612 to G-2612), which resulted in the amino acid exchange of His (CAC) to Arg (CGC), as has been recorded for the *uzu* variant in six-rowed barley [93]. 

Recently, two mutations were identified, using the TILLING approach, within *HvBRI1*, the barley homolog of the Arabidopsis *BRI1* gene [98]. Two mutant lines that carry different mutations in the kinase of the HvBRI1 protein are *M6649*, which has the serine to asparagine (S1098N), and *M6945*, which has arginine to lysine (R953K), substitution, respectively. The homozygous mutants of both lines exhibited a considerable reduction in plant height and spike length when compared to the wild-type HTX. In addition, *M6649* had fewer grains per spike than *M6945*, which was more comparable to the wild variety because of that trait. The production of semi-dwarf barley varieties will benefit from the addition of these mutants to the current genetic resources.

### 3.5. Bd21 Allele of Brachypodium

A mutant *Bd21* line (BdAA900) was discovered in the Brachy-TAG collection that had a T-DNA insertion in the *Brachypodium distachyon* gene called *Bradi2g48280* [99]. It is strongly suggested that *Bradi2g48280* is a *BRI1* homolog, since the expected Bradi2g48280 polypeptide shares 90% amino-acid homology with the barley BRI1 protein and 84% with the D61 rice receptor. A severe dwarf and twisted plant phenotype was displayed by the T-DNA insert, indicating the existence of a single T-DNA locus. When compared to wild-type plants, mutant plants were found to be considerably less responsive to the application of brassinosteroid using a leaf-unrolling assay. 

### 3.6. mtbri1 Alleles of M. truncatula

Three independent *Tnt1* insertion mutant lines were isolated and molecularly characterized in the *Medicago truncatula Tnt1* insertion mutant population named *mtbri1-1*, *mtbri1-2*, and *mtbri1-3* of *MtBRI1*gene [54]. According to sequence alignment, *Tnt1* inserts in the three mutants at positions 709, 15, and 468 bp downstream of the initiation codon, respectively (Figure 2). *MtBRI1* mutations disrupt the BR signaling system, resulting in developmentally abnormal phenotypes that are characteristics of *bri1* mutants. Despite the varied sites of *Tnt1* inserts in *MtBRI1*, *mtbri1* mutant alleles exhibit identical defective phenotypes, including acute dwarfism, compact shoots with curled dark-green leaves, shortened petioles and leaf blades, and reduced lateral roots. The *mtbri1* seedlings that grew in the dark display characteristic photomorphogenesis, displaying short hypocotyls and open cotyledons. Furthermore, in contrast to pink nodules in wild-type plants, nodules in *mtbri1* mutants are smaller and primarily white, and are deficient in nitrogen fixation, representing a novel observation in *bri1* mutants. In pea, there is no change in nodule size or nitrogen fixation activity among the wild-type variety and BR response mutant *lka* [83]. 

### 3.7. E29 Allele of Pepper

A dwarf pepper mutant, *E29,* which is weakly insensitive to BR, was isolated from EMS mutagenesis screening of the pepper-inbred line 64 [100]. The E29 mutant displayed a short-statured, compact plant with wide, thick, dark-green leaves. The stems’ histological longitudinal sections showed that, in *E29*, the cell width had increased but the cell length had decreased. When comparing *E29* to 6421, there was a 74.7% decrease in the fruit production per plant, due to a reduction in the number of flowers. Only one recessive gene produced the mutant phenotype, and inside the kinase domain of CaBRI1 there was an allelic mutation. An amino acid at position 1157 (Pro1157Ser) of CaBRI1 changed from proline to serine as a result of the C-to-T mutation (Figure 2). Because Pro1157Ser is highly conserved amongst homologous proteins in the kinase domain, a mutation in CaBRI1 reduced its kinase activity. BAK1 cannot be activated by kinase-dead BRI1 and, as a result, the BR signal cannot be transmitted downstream [23,101]. The level of BL in the *E29* mutant was significantly higher than that of 6421 (3.0-fold) similar to the high levels of BL that were recorded in Arabidopsis mutants *bri1-4*, *bri1-5*, *bri1-6* [64] and the pea mutant *lka* [83,84].

## 4. Biological Uses of *bri1* Alleles and Its Orthologs

**Figure 5:**
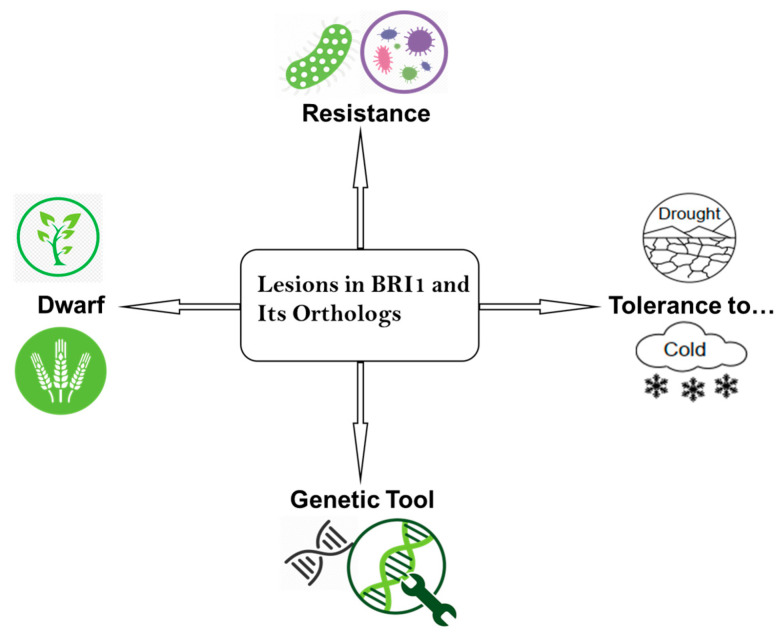
Showing biological uses of *bri1* alleles and its orthologs. These mutants have some unique features, i.e., resistance to a range of pathogens, tolerance to cold and drought; agronomically valued dwarf variety, and as a genetic tool.

*bri1* and its orthologs offered some valuable unique features. Weak *bri1* alleles are excellent genetic tools that have been extensively employed to investigate the role of brassinosteroid and its receptor BRI1 in plant growth and development. These mutants also aided in the discovery of many key proteins of the BR signaling pathway. Its orthologs in the plant kingdom have offered agronomically valuable traits that have been used to develop better plant varieties (Figure 5).

### 4.1. bri1 Alleles as a Genetic Tool

The use of many of the early identified *bri1* alleles in large-scale genetic transformation experiments is limited, since they are strong alleles with significantly lower male fertility [51]. Conversely, fertile weak *bri1* alleles, like *bri1-5* and *bri1-9*, are powerful genetic tools employed for exploring the complete BR signaling pathway through extragenic modifier screens [64]. Our understanding of the BR signaling cascade, from BR perception to downstream responding-gene regulation, has greatly benefited from identifying the essential regulatory components using weak *bri1* mutants [51]. For instance, activation-tagging genetic screens employing *bri1-5* as background revealed several important elements controlling BR signal transduction or BR homeostasis, such as BRS1 [102], BAK1 [30], and BSU1 [32], BRL1 [28], BEN1 [103], and TEOSINTE BRANCHED1/CYCLOIDEA/PROLIFERATING CELL FACTOR1 (TCP1) [104]. The Dof-type transcription factor COG1 is an activation-tagged genetic modulator of *bri1-5* that controls BR biosynthesis by upregulating PHYTOCHROMEINTERACTING FACTOR 4 and 5 (PIF4 and PIF5) transcription [105]. From ethyl methanesulfonate (EMS)-mutagenized *bri1-9*, a suppressor screen was carried out to isolate EBS1 to EBS7, a group of functionally associated proteins controlling endoplasmic reticulum (ER) quality regulation [52,106,107,108]. Using an EMS-mutagenized *bri1-119* library, a suppressor screen was used to identify BES1, a crucial downstream transcription factor involved in the BR signaling pathway [40]. Since its identification and characterization, *bri1-301* has been frequently used as a genetic tool and an additional weak *bri1* allele. Two families of atypical bHLH proteins, i.e., ATBS, and AIF1, capable of controlling BR signaling, were identified via activation-tagging the genetic suppressor of *bri1-301* [109].

### 4.2. Dwarfism: An Important Trait for Agriculture

One of the most important characteristics of crop breeding is dwarfism. High-yielding semi-dwarf rice (*Oryza sativa*) and wheat (*Triticum aestivum*) cultivars, along with heavy-nitrogen fertilizer applications, were the key factors that made the Green Revolution possible [110]. Dwarf mutants are highly significant in many plant species and have been extensively studied because of their agronomic significance. Gibberellin (GA), a phytohormone, is one of the key elements linked to the dwarf phenotype. It is worth noting that two Green Revolution genes from rice *semidwarf1* (*sd1*) and wheat *Reduced height1* (*Rht1*) are associated with GA biosynthesis and signaling, respectively [111,112,113].

In contemporary crops like barley, the fourth most abundant cereal in terms of area and cultivated tonnage, mutant-based breeding approaches for optimizing brassinosteroid metabolism and signaling pathways might enhance lodging behavior [114]. Barley was shown to be a key crop for creating high-yielding semi-dwarf varieties. Since the *uzu* gene showed lodging resistance in semi-dwarf barley accessions, the *uzu* gene has been inserted into all hull-less barley grown in Japan [115]. The missense mutation of *HvBRI*, a counterpart of Arabidopsis *BRI1*, is the cause of the *uzu* phenotype, which is brassinosteroid-insensitive [93,115,116]. This demonstrates that a BR-related mutation is a viable target for generating high-yielding semi-dwarf cultivars. The *uzu* allele has been cultivated for more than a century in East Asia and is currently found in winter barley varieties in Japan, the Korean peninsula, and China [115]. Its agronomic significance arises from the sturdy and short culm that offers resistance to lodging and an erect plant architecture that can withstand dense planting. Despite the long history of *uzu* mutation in barley breeding, it was only found in winter barley varieties in Northeast Asia [115]. The *uzu* allele is sensitive to high temperatures, which may be the reason this haplotype did not spread around the globe during the era of the Green Revolution. For culm-length regulation in barley breeding, additional alleles of *HvBRI1*, particularly *uzu1.256* with its milder phenotype and lack of *uzu*’s strong temperature sensitivity, could be taken into consideration as a more reliable alternative [94]. The semi-dwarf phenotype of the *093AR* mutant, which is a result of a mutation in the *HvBRI1* gene, was not accompanied by a decrease in fertility or seed output obtained from the European spring barley germplasm. When given large doses of fertilizer, semi-dwarf varieties of cereal are more resilient to lodging under unfavorable conditions; hence, the mutant may be the foundation of semi-dwarfism for barley breeding [95]. Two-rowed Chinese landrace ‘TX9425′, containing a mutation the same as the semi-dwarf *uzu* gene in six-rowed barleys, showed agronomically valuable traits that provide diagnostic markers for the selection of genes for semi-dwarf stature in barley breeding programs [97].

Morinaka et al. [88] picked the *d61-7* line of dwarf rice, which has a unique *d61* allele. Because of its agronomically advantageous characteristics, such as semi-dwarf stature, upright leaves, and elongated neck internodes, it has been chosen for further analysis into grain production in rice fields. The *d61* mutant’s erect-leaf phenotype may be used to create rice varieties that yield effectively at high planting densities, as seen by the enhanced grain production of *d61-7* in field trials, even at high densities. The tiny grain of *d61-7*, however, counters the rise in agricultural productivity; for example, at high planting density, the weak mutant allele *d61-7* increases biomass by 35% relative to the wild type, but due to the small grain size of *d61-7*, there is no change in grain yield. 

Recently, a new allele *E29* of the height-regulating gene *CaBRI1* was obtained by EMS mutagenesis of the pepper inbred line 6421. This variant has theoretical and practical implications for plant breeding suitable for facility cultivation and controlled pepper-variety harvesting [100], which makes it an excellent parent material when generating new dwarf cultivars.

### 4.3. Pathogen Resistance

Plants are exposed to a variety of pathogens in their natural habitat, and pathogens can substantially affect plant growth; in response, plants have developed many mechanisms to combat these pathogens. BRs are actively involved in responses to diverse pathogen attacks including viruses, bacteria, fungi, and nematodes [117]. The impact of *BRI1* mutations on disease resistance to a range of pathogens has not been studied to a great extent; however, the *uzu* allele of barley resistance to a variety of pathogens is well documented. Because the *HvBRI1* mutation increased resistance to a wide variety of harmful microbes, despite heterogeneity in their infection and virulence methods, BR signaling functions antagonistically with basal defense systems [56]. 

The *BRI1* mutation alleviated disease induced by *M. oryzae* in both *Brachypodium distachyon* and barley. [56]. Disruption of the BR signaling system has been associated with reduced tolerance to abiotic stress, yet the lack of a biotroph–necrotroph trade-off in both *B*. *distachyon* and barley *bri1* lines shows that introducing this mutation may be beneficial in plant breeding. Increased resistance to hemibiotrophic and necrotrophic fungal infections with a brief biotrophic phase results from decreased BR sensitivity. The *BRI1* mutation in barley does not suffer disease resistance, in contrast to the *Rht* and *Sln1* GA-insensitive alleles [56]. The barley *uzu* mutation showed increased resistance to various diseases: eyespot disease of stems caused by *Oculimacula* spp.; take-all of roots triggered by *Gaeumannomyces graminis* var. tritici; and crown rot disease within the stem caused by *Fusarium fungus* [56,118].

With the help of genetic techniques, Chen and colleagues [118] discovered that barley *uzu* (*Hvbri1*) lines exhibit greater resistance to fusarium crown rot (FCR) than their tall counterparts, suggesting that decreased BR signaling amplifies resistance to *Fusarium* spp. Ali et al. [119] employed a blend of transcriptome and biochemical analyses to ascertain the defense-related differences between *uzu* derivatives and their parent barley genotypes. Comparing *uzu* derivatives to their parental lines, they demonstrated greater resistance against the obligate pathogen Barley Stripe Mosaic Virus (BSMV), the necrotrophic net blotch pathogen *Pyrenophora teres*, and the toxic hemibiotrophic fungus *Fusarium culmorum*, which causes Fusarium head blight (FHB, also referred to as scab disease of cereals). An unknown pathway may be responsible for the pleiotropic effects of *BRI1* in the *uzu* resistance.

### 4.4. Stress Tolerance

BRs are shown to play a major role in plant responses to stresses such as cold and drought [120], which are two major issues to be addressed due to climate change. Designing and breeding stress-tolerant agricultural cultivars should be the main goal in contemporary breeding efforts, considering the current variations in the climate [121,122].

#### 4.4.1. Drought

Drought stress was applied to some of the semi-dwarf *HvBRI1* gene mutants, such as the *uzu1.a* mutant. When compared to the wild-type cultivar, the semi-dwarf BR mutants showed delayed wilting in response to drought stress [123]. To clarify the effect of BR signaling disruptions on the build-up of non-enzymatic antioxidants in both control and drought conditions, the semi-dwarf mutants of the *HvBRI1* gene were examined. Remarkably, the glutathione accumulation study showed that the BR-insensitive mutants had far smaller amounts of this antioxidant under control circumstances as compared to the BR-biosynthesis mutants and the wild-type cultivar. Consequently, it was concluded that for normal accumulation of glutathione, BR sensitivity is essential in barley [124].

#### 4.4.2. Cold

A significant risk to plant growth and development, as well as effects on plant distribution and crop yield, is posed by cold stress. Kim et al. [125] reported that *bri1* mutants insensitive to brassinosteroids are more resilient to cold stress compared to the wild type. *bri1* mutants are reported to accumulate larger quantities of BRs in the cell [64], and *bri1-9* which is deficient in BR signaling, leading to dwarfism, has a stronger resilience to cold compared to the wild-type, whereas *BRI1*- overexpressing transgenic plants are far more sensitive to cold. Compared to either wild-type or *BRI1*-overexpressing plants, *bri1-9* showed higher levels of endogenous expression of a combination of stress-inducible genes and genes expressing transcription factors that promote the function of stress-inducible genes. Even with the lack of a pathogen attack or environmental challenges, disease-resistant genes specific for numerous pathogens and genes expressing proteins for abiotic conditions were elevated in the *bri1-9* mutant. This gives agriculture-based important information on how much endogenous growth retardation is acceptable to compensate for decreased growth associated with adverse environmental circumstances.

The isolation of mutants possessing favorable phenotypic features (e.g., semi-dwarfism, erect stature) in modern agricultural operations are needed. In certain situations, these characteristics also increase resistance to environmental challenges, making high-density planting possible [126]. Given the continued effects of global climate change, creating such grain cultivars appear to be especially crucial [126].

## 5. Conclusions and Future Perspectives

Genetic strategies like mutant screens identified several essential players in BR production and signaling and illustrated the significance of BRs in plant growth [127]. The present understanding of the BR signaling system has been substantially improved by the *bri1* alleles. *bri1* alleles are excellent genetic tools that can be used to discover more components of the BR signaling pathway and fill in the gaps in brassinosteroids’ crosstalk with other phytohormones. One of the well-studied hormone receptors in plants is BRI1; however, *bri1* alleles may be very helpful in examining it to learn more about its functions in plant development, growth, and stress-related responses. 

To date, in addition to the model plant Arabidopsis, the BR signaling pathway has been explored to a somewhat greater extent in rice [128]; nevertheless, knowledge of the mechanism of BR signaling in important cereal crops is still limited. Consequently, *bri1* allele orthologs in respective plants can be valuable as genetic resources to uncover further elements of the BR signaling pathway in numerous significant cereal crops. Furthermore, identification and mutational analysis of BR signaling components in these agronomically valuable cereal crops could help to produce semi-dwarf phenotypes with the desired qualities to be produced in high densities. 

The plant yield of barley has been successfully increased by the BR-related *uzu* mutant [94]. This is an illustration of a chosen genetic variety that has only slightly modified the stature of the plant, but that modification has proven to be advantageous for both productivity and environmental adaption. Even more encouraging is the fact that *uzu* barley also showed improved pathogen resistance [119]. Modern genome techniques, such as genome editing and molecular breeding, have made it possible to purposely introduce these mutations into a range of plants to create improved agronomic varieties. These revelations can provide crucial understanding and insights to design strategies for producing crops with improved attributes.

Climate change is one of the core issues faced by the human population around the globe. The production and distribution of a particular crop may be affected by modifications to its growth environment brought about by climate change. Scientists are under demand to create crop varieties that can withstand extreme weather due to climate change; *bri1* orthologs might serve as the ideal genetic resource to create such crop varieties.

Recently, there has been a rapid development of single-cell sequencing techniques for examining the complex environment of individual cells. These technologies can be used to study the BR signaling pathway in the model plant Arabidopsis and other important crops to obtain pieces of information for the development of improved plant varieties. 

## Figures and Tables

**Table 1:** *bri1* alleles in Arabidopsis.

Alleles	Base Pair Change	Amino Acid Change	Accession	Allelic Strength/ Phenotype	Possible Mechanism	References
*bri1-1*	G2725A	Ala-909-Thr	Col-0	Strong	Extremely weak in vivo BL-stimulated BAK1 phosphorylation	[49,61]
*bri1-2/cbb2*	Transposon insertion	Nil	Col-0	Strong	Impaired BRI1	[63]
*bri1-3*	4-bp deletion after 2745	STOP 44 aa downstream	Wassilewskija-2 (WS2)	Strong	Impaired BRI1 KD	[64]
*bri1-4*	10-bp deletion after 459	STOP 13 aa downstream	WS2	Strong	No BRI1	[64]
*bri1-5*	G206A	Cys-69-Tyr	WS2	Weak	ER retention	[64]
*bri1-5R1*	G260A	Gly-87-Glu	*bri1-5*/WS2	Weak	Partially restore ER retention of *bri1-5*	[65]
*bri1-6/119*	G1931A	Gly-644-Asp	Enkheim-2 (En-2)	Weak	Unknown	[49,64]
*bri1-7*	G1838A	Gly-613-Ser	WS2	Weak	Impaired BL binding	[64]
*bri1-8/108/112*	G2948A	Arg-983-Gln	WS2/Col-0	Intermediate	Autophosphorylation cannot be detected in vitro	[49,64]
*bri1-9*	C1985T	Ser-662-Phe	WS2/Col-0	Weak	ER retention	[64]
*bri1-101*	G3232A	Glu-1078-Lys	Col-0	Strong	Autophosphorylation cannot be detected in vitro	[18,49]
*bri1-102*	C2249T	Thr-750-Ile	Col-0	Strong	Unknown	[49]
*bri1-103/104*	G3091A	Ala-1031-Thr	Col-0	Strong	Unknown	[18,49]
*bri1-105/106/107*	C3175T	Gln-1059-Stop	Col-0	Strong	Impaired BRI1 KD	[18,49]
*bri1-113*	G1832A	Gly-611-Glu	Col-0	Strong	Impaired BL binding	[18]
*bri1-114/116*	C1747T	Gln-583-Stop	Col-0	Strong	No BRI1	[18,49]
*bri1-115*	G3143A	Gly-1048-Asp	Col-0	Strong	Unknown	[18]
*bri1-117/118*	G3415A	Asp-1139-Asn	Col-0	Strong	Unknown	[18,49]
*bri1-120/cp3*	T1196C	Ser-399-Phe	Landsberg erecta	Weak	Unknown	[66]
*bri1-201*	8bp deletion	STOP 44 aa downstream	Col-0	Strong	Premature stop	[67]
*bri1-201-1*	G1831A	Gly-611-Arg	WS2	Strong, late-flowering *ld-3* enhancer	Unknown	[8]
*bri1-202*	C2854T	Arg-952-Trp	WS2	Strong, late-flowering ld-3 enhancer	Unknown	[8]
*bri1-235*	C468T	Ser-156-Phe	Col-0	Weak	ER retention	[68]
*bri1-301*	GG2965/6AT	Gly-989-Ile	Col-0	Weak	Kinase dead, thermally unstable	[69,70,71]
*salade*	Transposon insertion	genome deletion	Col-0	Strong	No BRI1	[72]
*bri1-701*	T-DNA insertion	Nil	Col-0	Strong	No BRI1	[73]
*bri1-702*	C3148T	Pro-1050-Ser	Col-0	Weak	Reduced autophosphorylation in vitro	[51]
*bri1-703*	G3166A	Glu-1056-Lys	Col-0	Strong	Autophosphorylation cannot be detected in vitro	[51]
*bri1-704*	G3079A	Asp-1027-Asn	Col-0	Strong	Autophosphorylation cannot be detected in vitro	[51]
*bri1-705*	C2156T	Pro-719-Leu	Col-0	Subtle	Disrupt the formation of hydrogen bonds among BRI1, BL, and BAK1	[51]
*bri1-706*	C758T	Ser-253-Phe	Col-0	Subtle	Unknown	[51]
*bri1-708*	C2947G	Arg-938-Gly	Col-0	Strong	Autophosphorylation cannot be detected in vitro	[51]
*bri1-709*	G2543A	Trp-848-Stop	Col-0	Strong	Premature stop	[51]
*bri1-710*	G1858A	Gly-620-Arg	Col-0	Subtle	Unknown	[51]
*bri1-711*/*bri1^cnu4^*	G2236A	Gly-746-Ser	Col-0	Subtle	Unknown	[51,74]
*bri1^cnu1^*	G2831A	Gly-944-Asp	Col-0	Weak	Unknown	[75]
*bri1^cnu3^*	G2307T	Arg-769-Trp	Col-0	Weak	Unknown	[74]

**Table 2:** *d61* alleles of rice.

Allele	Base Pair Change	Amino Acid Change	Phenotype	Fertility	Reference
*d61-1*	C to T	Thr-989-Ile	Mild	Fertile	[55]
*d61-2*	G to A	Val-491-Met	Intermediate	Fertile	[55]
*d61-3*	A to C	His-420-Pro	Severe	Sterile	[89]
*d61-4*	G to T	Glu-847-Stop	Severe	Sterile	[89]
*d61-5*	A to T	Asn-426-Tyr	Severe	Sterile	[89]
*d61-6*	2 bp insertion	Asp-759-stop	Severe	Sterile	[89]
*d61-7*	C to T	Ala-467-Val	Mild	Fertile	[89]
*d61-8*	G to A	Gly-522-Glu	Mild	Fertile	[89]
*d61-9*	G to A	Gly-539-Asp	Mild	Fertile	[89]
*d61-10*	C to A	Thr-854-Ile	Mild	Fertile	[89]
*Fn189*	A to T	Ile-834-Phe	Mild	Fertile	[90]
